# Overabundant milk supply: an alternative way to intervene by full drainage and block feeding

**DOI:** 10.1186/1746-4358-2-11

**Published:** 2007-08-29

**Authors:** Caroline GA van Veldhuizen-Staas

**Affiliations:** 1 Private practice, Merkelbeek, The Netherlands

## Abstract

**Background:**

Too much or too little milk production are common problems in a lactation consultant's practice. Whereas underproduction is widely discussed in the lactation literature, overabundant milk supply is not. In my practice I work with women who experience moderate to severe oversupply syndrome. In most cases the syndrome can be successfully treated with full removal of milk followed by unilateral breastfeeding *ad lib *with the same breast offered at every breastfeed in a certain time block ("block feeding").

**Case presentations:**

Four cases of over-supply of breast milk are presented. The management and outcome of each case is described.

**Conclusion:**

Overabundant milk supply is an often under-diagnosed condition in otherwise healthy lactating women. Full drainage and "block feeding" offer an adequate and userfriendly way to normalize milk production and treat symptoms in both mother and child.

## Background

Breastfeeding is the method of first choice for feeding any infant. Both the World Health Organization (WHO) and many leading organizations of pediatricians, as well as many governments advise that children be exclusively breastfed for a half year from birth and continue to be breastfed in combination with suitable foods for an extended time after that [1]. Breastmilk production is an inborn capability in women, with only rare exceptions due to anatomical or physiological pathology. Even in these rare cases, partial breastmilk production may sometimes be possible. Even though these exceptions are extremely rare, a widespread belief exists that many women are not capable of producing enough, or good enough, milk for their children [2]. Methods to treat real or perceived low milk supply are well referenced in the literature [3]. Overabundant milk supply or hyperlactation on the other hand is not discussed in depth in the literature. There is no consensus on treatment or terminology. However, overabundant milk supply can be as devastating for the continuance of breastfeeding as underproduction. In this paper, I propose a definition, etiology and a possible "intervention" which has been found to be more effective in my practice than other common breastfeeding management solutions.

### Definition of overabundant milk supply

In day-to-day language the problem of "too much milk" is mostly referred to as overproduction, overproduction syndrome or overabundant milk supply. In the professional literature the word hyperlactation is also used, but is linked to different descriptions [4-7]:

1. Overactive production by the milk producing glands during lactation. This is also referred to as overproduction or overproduction syndrome;

2. Milk production in a non-lactating woman or in a man, also referred to as galactorrhea. Some references use galactorrhea also in definition 1;

3. Continuance of lactation beyond the normal period.

The focus of this paper is the first definition: an overabundant supply of milk in an otherwise healthy lactating woman.

### Physiology

In physiological lactation fullness of the breast and galactostasis (milk remaining in the breast without removal) will lead to a decreased milk production. The accumulation of milk in the breast will reduce the binding of prolactin to its membranes. This will happen in any breast that gets overfilled, independent of the status of the other breast. The reduction in the binding of prolactin to membrane receptors will create an inhibitory effect on levels of milk production. In full alveoli lactocytes (milk producing cells) will have a lowered uptake of prolactin from the blood. If the full breast is emptied, prolactin again will bind to the membrane receptors, thus enhancing milk synthesis. The more empty the alveoli, the higher the milk synthesis rate, slowing down as the breast refills [8-10]. Evidence exists that there are two interacting mechanisms regulating the rate of milk synthesis. The first involves the Feedback Inhibitor of Lactation (FIL). "FIL is an active whey protein that inhibits milk secretion as alveoli become distended and milk is not removed. Its concentration increases with longer periods of milk accumulation, down regulating milk production in a chemical feedback loop. The inhibition of milk secretion is reversible and dependent on concentration; it does not affect the composition of the milk because it affects the secretion of all milk components simultaneously" [3] [p. 76]. FIL has been identified as a small protein synthesised by the secretory epithelial cells (lactocytes) that accumulates within the alveolar lumen along with other milk constituents. However as FIL is an autocrine inhibitor of milk synthesis, milk synthesis declines as FIL accumulation within the lumen increases. When milk is removed from the breast the concentration of FIL declines and milk synthesis once again increases. The other mechanism involves the interaction of lactocytes with the basement membrane to which they are attached. It is hypothesised that as the breast fills with milk, the shape of the lactocytes changes such that the prolactin receptor is deactivated and milk synthesis is slowed, and eventually ceases [11]. Although the FIL and cell shape mechanisms likely act independently to influence the activity of prolactin upon the breast, the interaction and interplay of these two factors upon milk synthesis has yet to be fully elucidated.

### Description and etiology

Hyperlactation can be caused by breastfeeding mismanagement, hyperprolactinemia or congenital predisposition. Livingstone defines hyperlactation as "mother and child hyperlactation syndrome", because in both mother and child, symptoms can lead to pathology [7]. The hyperlactating woman will often experience a constant feeling of (over-) fullness, engorgement and tension. She may leak milk in between feedings, or leak copiously from the opposite breast during feedings, and has an increased risk for mastitis.

The infant may appear to be a greedy feeder, struggling not to choke or aspirate milk. He or she may often spit up after feedings and/or have reflux-like symptoms, and suffer from intestinal gas, colic and explosive, often green and foamy stools. The baby may show either a very low or a very high weight gain. The baby's struggle to cope with rapid flow may result in restless nursing behaviour, or even aversive behaviour, such as breast refusal or shortened feeds. Fussiness, crying and possible low weight gain can lead the mother to think that her milk is insufficient in quantity and/or quality.

An infant drinking from an overproducing breast may not be able to empty the breast far enough to obtain the fatter milk that is available in the more empty breast. The relatively high sugar, but low fat content of the diet may cause rapid gastric passage, which may lead to lactose concentrations in the small bowel too high for the infant's lactase potential, resulting in frequent diarrheic bowel movements [12].

A common secondary symptom in hyperlactation syndrome is a sub-optimal nursing technique in the infant. This may be the result of the child's attempts to cope with an overabundant milk flow, sometimes slipping from an optimum latch in order to clamp down on the nipple to slow the flow, often traumatizing the mother's nipple in the process. Or the infant may develop a conditioned habit of drinking but passively suckling at a breast that will give milk without any effort by the infant itself. This has the potential to lead to supply problems after 4–6 weeks when supply stimulation patterns transition from primarily hormonal stimulation to feedback inhibition mechanisms.

### Usual treatments

A treatment for overproduction commonly mentioned by lay advisors is to pump some milk directly prior to breastfeeding [13,14]. Rationale for this option is to stimulate the surge of the milk ejection reflex and allow the peak of the overwhelming milk flow to pass, remove some of the lower-fat foremilk and to enable the child to receive the fat-rich hindmilk sooner. An argument against this approach is that frequent pumping in combination with normal breastfeeding will increase milk production and eventually increase the problem. Many professionals are now adopting an evidence-based approach to reduce milk supply by enhancing breastfeeding management. Wilson-Clay and Hoover follow Livingstone in advising to not decrease feeding frequency, but to decrease the number of times that the child will be offered "the other breast" [15]. They advise restricting breastfeeding at one breast for a set number of feedings or a set amount of hours. The rationale for this is to produce a relative galactostasis in the temporarily unused breast, leading to a reduction of milk production due to the accumulation of the feedback inhibitor of lactation (FIL) and the change in lactocyte shape, both of which lead to a decline in the rate of milk synthesis. This practice will need close observation for signs of plugged ducts and mastitis. The difficulty in this approach is that the total pool of accumulated milk is reabsorbed very slowly. This pattern, if not carried out gradually and carefully, risks plugged ducts, discomfort and potential mastitis for the mother and may force the baby to continue coping for a long time from the start of the treatment. Berghuijs, describing complete drainage of both breasts as a treatment for hyperlactation, presented a significant modification of this method in a case discussion [16]. She introduced the term "milk lakes" to describe the cumulative production and storage of milk due to the combination of overactive milk production, inter-feeding leakage and inefficient drainage by the infant. Berghuijs advises complete mechanical pumping followed immediately by unrestricted bilateral feeding. In her view the accumulated milk is the problem and frequent bilateral feeding will regulate milk production after the "milk lakes" have been cleared. This method does not use FIL to decrease milk production. Bilateral stimulation at every feeding session may lead to a very fast increase in milk production and new filling of the "milk lakes." The Berghuijs approach however did lead to the development of the method presented in this article where emptying the breast is followed by a way to use FIL in order to decrease milk production to more desired levels. Pharmaceutical treatment, for instance with pseudoephedrine or estrogen-containing contraception is another possibility sometimes mentioned [17]. Both of these can lead to unwanted side-effects. Naturopathic therapists tend to advise *Salvia officinalis *(the culinary herb sage) as a tincture or concoction [18]. Sage is a powerful lactation inhibitor and should be used with caution if the aim is to decrease supply, rather than lactation suppression.

What is most needed is to find a way to quickly reduce the excess of milk without increasing milk production. Most remedies focus on this reduction side while failing to take into account the infant's side of this syndrome. The optimal treatment would thus also include a way to ensure comfortable nursing for the baby, without having to drink against an overactive milk flow and without interfering with physiologic gastroenteral function. In overproducing mothers the baby tends to receive a relative overload of lactose and a relative shortage of fat. Normal gut function needs a balance between carbohydrates and fat in the food that is being digested.

In my private practice, I have developed this combination of remedies into one that I call the full drainage and block feeding method (FDBF).

### Description of FDBF

The treatment sequence starts with an as-complete-as-possible mechanical drainage of both breasts. It is impossible to really empty an active, lactating breast completely, because the production of milk is an ongoing process. Emptying the breast is a major trigger for renewed production activity. Manual expression is a possibility, too, but in most cases mechanical extraction will work more efficiently and rapidly, especially if a simultaneous double pump is used. The infant will latch on immediately after drainage and will be offered both "empty" breasts to satisfaction. Many infants will fall asleep fully satisfied with high fat hindmilk, many for the first time. Subsequently the rest of the day is divided into equal time blocks starting with about three hours, initially. Every time the infant shows hunger cues or other signs of interest in the breast the same breast will be offered without any restriction in either frequency or duration of feeds. At the end of such a time block, or after a multi-hour period of sleep, baby will be offered the other breast for all feeds within the next time block. It is important that the best possible positioning and efficient latching techniques be used starting right from the very first feeding after pumping, for the sake of both the baby's improved suckling habits and the mother's comfort and future production. Depending on the seriousness of the symptoms time blocks may gradually be increased to 4, 6, 8 or even 12 hours. For less complex situations one-time mechanical drainage will suffice; for others occasional repetition may be necessary. Intervals between drainage will gradually increase as the symptoms lessen.

Mothers must be cautioned not to drain the breasts too often in order to avoid extra stimulation for milk production. Only if engorgement is becoming severe again should another drainage be carried out. In using FDBF the mother will need to be instructed, cautioned and monitored for temporarily recurring over-fullness and plugged ducts or mastitis. After the first full drainage, in some women the breasts will initially continue to produce more than asked for and thus refill. In many others just a single full drainage will suffice to decrease milk production to acceptable levels.

## Case presentations

### Case 1

Mrs. B is a healthy mother of a toddler and a nine day old infant. She is breastfeeding both children. With her first child she experienced oversupply syndrome during the first four months postpartum. Treatments included milk removal prior to breastfeeding to soften the breasts, unlatching the child at the start of milk ejection reflex to release the most powerful milk flow, and stretching feeding intervals to ease baby's stomach. After four months of trying these strategies milk production stabilized at an acceptable level. In the first eight postpartum days with the second child she was advised to use the same strategies, with no effect until her visit at the lactation consultant's practice on day nine. Baby started to refuse feeding at the breast or only wanted to breastfeed lying down. Baby was fussy and showing signs of stomach problems. Breasts remained full and uncomfortable in between feeding sessions. One health care provider urged her to stop tandem nursing; this advice was not an option for this mother and toddler. Mrs. B does not want to experience oversupply for four months this time. Assessment showed an apparently healthy, well gaining baby and toddler, nipples within the range of functional-normal with no signs of damage and no oral cavity abnormalities. Baby latched on well, but fussed while drinking, losing vacuum intermittently. Mrs. B reported that the infant had multiple very wet diapers daily and copious loose yellow stools.

FDBF was discussed with Mrs. B. She decided to try this, despite some questioning how this would fit into tandem nursing. She started expressing both breasts as completely as possible the same day, then putting the infant to the breast. At observation, the baby nursed well, without losing vacuum or fussing. He fell asleep after finishing the second breast. Mrs. B started block feeding after this initial milk expression and subsequently breastfed *ad lib *unilaterally in blocks of three hours. The toddler was nursed within the block schedule that was set for the infant and did nurse well. During the first 24 hours her breasts started filling again and she repeated expressing 30 hours after the initial expression. Block feeding continued as started. In the course of the following week Mrs B. had to express one more time after 72 hours, while continuing a three hour block feeding schedule. At follow up at one month postpartum Mrs. B reported no more signs of overproduction. The toddler kept nursing occasionally, following the infant's schedule.

### Case 2

Mrs. A. is breastfeeding her eight day old healthy boy, one breast per feeding session. Baby is fussy and noisy at breast, and Mrs. A reports that she can "hear the milk squirt into baby's stomach". Baby needs to burp often, but this does not ease his stomach-ache. Baby is not happy, is colicky and often brings up substantial amounts of milk after feeding. Stools are greenish, "foamy" and come often and in large amounts. Baby's weight is 310 grams above birth weight on day 8, without initial weight loss. Previously health care providers diagnosed mother and baby with overproduction syndrome and advised Mrs. A to hand-express some milk prior to feeding, breastfeeding while lying on her back, and block feeding; another provider advised to stretch feeding intervals to ease baby's stomach. These approaches did not work for Mrs. A. and she wants to stop breastfeeding because she cannot cope with this situation. Visiting the lactation consultant's practice is a last resort.

Breast assessment showed rather small, but full and firm breasts. Mrs. A does not report any pain, but she is in substantial discomfort during most of the day and night. Her concern, however, is more about baby's apparent unhappiness and pain. After discussion of FDBF, Mrs. A started with initial expression of her breasts as completely as possible; 150 mls was expressed from the left breast and 200 mls from the right breast. The upcoming 24 hours were divided into blocks of three hours. Special attention was paid to informing Mrs. A. that babies will know how much milk they need per session and that there is no need to urge her child to drink more than he wants at a time. This resulted in more frequent, smaller feedings. Mrs. A's breast did refill in the first 24 hours, but not so much that repeated expression was needed. The milk ejection reflex remained strong, but the smaller amount of milk seemed to make it easier for the child to cope. The first feeding of every new block did give some discomfort in the first days, but baby's fussiness and colic disappeared.

### Case 3

Mrs. S is a healthy, 34 year old mother of a fifth child, 4 days postpartum. She breastfed her previous children without problems or complications. This fifth child was bigger than her previous children (4.530 kg), and he broke his clavicle during birth. He was supplemented with 20 mls of 10% glucose within two hours of birth and kept having low blood sugars. Mrs. S. decided to breastfeed him very frequently in order to prevent another supplementation and to increase his blood sugar levels. Baby was with his mother for the next 48 hours without more separation than needed for a bathroom visit and he was at the breast most of that time, frequently changing from one breast to the other. Blood sugar levels rose quickly and stayed high and stable. During the third postpartum day copious transitional milk came in and amounts were rising throughout the next 24 hours. Mrs. S. became painfully engorged and the baby began fussing at breast, returning significant amounts of milk. The breasts were hard to the touch, red and shiny. FDBF is discussed and started during the consultation. Mrs. S expressed a total amount of 500 mls and started block feeding in blocks of three to four hours. There was no need for further milk expression and milk production stayed within normal levels throughout a total lactation period of 30 months.

### Case 4

Mrs. D. has a normal figure with large breasts, cup size I. She is breastfeeding her two month old baby boy. Her initial engorgement did not decrease. The baby is breastfeeding frequently at short intervals, a few minutes at a time. Baby is not fussy at the breast, has no gastrointestinal problems and is growing within the higher range of normal. Breast assessment is difficult, because every handling of the breast causes milk to spray. Breasts do not feel very hard, but are firm and full. FDBF is discussed with Mrs D. and she starts milk expression during the consultation. She expresses 360 ml from the left breast and 340 ml from the right. Breasts do not feel much different to the touch, but Mrs. B does report a far less heavy feeling. After expressing, the day and night were divided into blocks of 4 hours initially, but this proved to be not working as expected. Full drainage was repeated the next day and the blocks were set at 6 hours. On the third day of intervention expression needed to be repeated and blocks were set at 12 hours. This at last did slow down milk production. Next milk expression followed 36 hours afterwards, and a final expression took place 48 hours after the last one. Mrs. D kept block feeding in blocks of 12, occasionally 24, hours. Attempts to shorten blocks to 6 hours led to increased milk volumes each time.

## Discussion

In some women it seems that the mechanism of regulation of milk production does not automatically work well. This can create the "milk lakes" Berghuijs mentions, and the ongoing production of more milk than needed. As thorough-as-possible drainage of the milk producing and storing systems and then paced demand to both breasts normalizes the systems of supply and demand. This normalization can work out rather quickly. The effects on the baby will show with the first feed after mechanical drainage. The infant will suckle without fussiness and will have the unfamiliar but pleasant experience of an immediate, gentle milk flow of double calorie, high fat milk that will not disturb coordination of his or her breathing and swallowing mechanisms and gastrointestinal tract symptoms or colic will quickly diminish. Overabundant milk supply is an often under-diagnosed condition in otherwise healthy lactating women. Symptoms can occur in both mother and child and may lead to pathology in both. Full drainage and block feeding offers an adequate and userfriendly way to normalize milk production and treat symptoms in both mother and child.

More research will need to be done to understand why some women will easily produce much more milk than needed and why for some it is so hard to regulate milk production to meet the needs of their children.

## Competing interests

The author(s) declare that they have no competing interests.
